# Dynamic platelet function: A novel biomarker in inflammatory arthritis?

**DOI:** 10.1371/journal.pone.0261825

**Published:** 2022-01-25

**Authors:** Eithne Nic an Riogh, Eimear Dunne, Sharon Cowley, Kelly Leamy, Geraldine McCarthy, Dermot Kenny, John Stack

**Affiliations:** 1 Rheumatology Department, Mater Misericordiae University Hospital, Dublin, Ireland; 2 Royal College of Surgeons in Ireland (RCSI), Dublin, Ireland; 3 School of Medicine, University College Dublin, Dublin, Ireland; Monash University, AUSTRALIA

## Abstract

**Background:**

Patients with inflammatory arthritis die prematurely of cardiovascular disease. Inflammation activates platelets. Since treatment of inflammatory arthritis is associated with reduced mortality, and decreased platelet reactivity reduces cardiovascular events, we hypothesised that platelet reactivity as measured by dynamic platelet function (DPF) would be increased in patients with inflammatory arthritis and that reactivity could be reduced with therapeutic intervention.

**Objectives:**

To characterise platelet function using a validated physiological assay in patients with inflammatory arthritis before and after disease improvement.

**Methods:**

22 patients were recruited and treated as per local protocol. DPF was measured at baseline and after clinical improvement. Video microscopy was utilised to measure dynamic platelet behaviour in microliters of blood perfused over von Willebrand factor (VWF) at arterial shear rates (1500 s-1). Motion-analysis software measured the number of platelets interacting with VWF, translocating across VWF, the speed and distance platelets travelled across VWF, and stably adhering to the surface. Platelet parameters at baseline and following improvement were compared using Wilcoxon signed rank test and paired student t-test. Changes in platelet function were correlated to inflammatory disease markers by Pearson Correlation.

**Results:**

18 patients completed the study. Platelet adhesion decreased and platelet motion increased following treatment. Tender joint count correlated with platelet adhesion (Pearson r = 0.616, p≤0.01) while CRP correlated with velocity of platelet movement (Pearson r = 0.563, p≤0.01).

**Conclusions:**

Improvement in clinical markers of inflammation is associated with a corresponding change in platelet function. Given the association between reduced mortality and decreased platelet reactivity our results suggest that an appropriate assay of platelet function could guide future therapy of patients with inflammatory arthritis.

## Introduction

Inflammatory arthritis is an umbrella term for rheumatologic conditions in which joints are inflamed. These conditions include rheumatoid arthritis, psoriatic arthritis, ankylosing spondylitis and seronegative spondyloarthropathies. It has been recognised that patients with inflammatory arthritis have increased cardiovascular risk and higher mortality rates compared to the general population [1]. Patients with these inflammatory conditions are exposed to greater levels of inflammatory cytokines which accelerate end organ damage and promote atherosclerosis. For example, rheumatoid arthritis patients have a prevalence of cardiovascular disease 50% higher than the general population and this represents their leading cause of mortality [2, 3]. This association with increased cardiovascular disease could be considered an extraarticular manifestation of inflammatory arthritis [4]. There is a compelling rationale to measure platelet function in inflammatory arthritis since platelet hyperreactivity is a potentially modifiable risk factor for cardiovascular disease in inflammatory arthritis.

Dynamic Platelet Function Assay (DPFA) uses the novel technology of motion analysis software to characterise platelet movement, surface coverage and interaction with von Willebrand factor (VWF). DPFA mimics the fundamental dynamic behaviour of thrombosis in vivo. VWF is immobilised on a coverslip that forms the base of a parallel-plate microfluidic flow chamber. Blood labelled with platelet-specific dye is perfused through the device and platelets are imaged by video fluorescence microscopy as they interact with the VWF surface. The MATLAB Image Processing Toolkit (version 7.12.0 R2001a) detects the position of each platelet in an x and y plane against a continuously changing background. It uses automatic thresholding of fluorescence microscopy images in microfluidics to determine platelet coverage and aggregate size distribution [5]. This innovative technology can accurately and rapidly screen clinical samples without the need for extensive operator training.

In contrast to assessing platelet aggregation whereby a single response to a single agonist is measured, this technology uses multiparameter analysis to assess a number of platelet motional parameters that result from platelet/surface-protein interactions. Previous studies have examined dynamic platelet function and aggregation in various cohorts including preterm neonates, pregnancy and malignancy and have shown that platelets in these cohorts behave differently to controls [6–8].

In this study we applied DPFA technology to provide information on the relationship between platelet function and activation in inflammatory disease during varying states of disease activity. We hypothesised that the treatment of inflammatory arthritis would reduce platelet reactivity and aggregation. To assess this we examined dynamic platelet function in patients with active disease and again following improvement in their inflammatory arthritis. We demonstrated that systemic treatment of arthritis reduces platelet interaction with VWF, the initiating event in thrombosis. This has the potential to affect the cardiovascular risk of this cohort.

We believe that results from this study and further analysis of platelet function in systemic inflammatory conditions will herald routine use of this technology to provide a rapid point of care measurement of thrombotic risk via surrogate marker of platelet activation.

## Methods

### Ethical approval

Ethical approval was obtained from the Mater Misericordiae University Hospital Institutional Review Board on the 19^th^ February 2018, Ref 1/378/1973. Patients or the public were not involved in the design, conduct, reporting or dissemination of our research.

### Patient recruitment

After written informed consent had been obtained, patients were recruited from an outpatient Rheumatology Clinic in the Mater Misericordiae University Hospital, Dublin. Patients were eligible if they had an established diagnosis of inflammatory arthritis, including rheumatoid arthritis, psoriatic arthritis or ankylosing spondylitis. Patients were required to be experiencing an active inflammatory episode defined clinically at the time of recruitment. For rheumatoid arthritis and psoriatic arthritis patients this was defined as the presence of swollen or tender joints. Of note, these patients all had a disease activity score (DAS-ESR or DAS-CRP) greater than 2.6. For ankylosing spondylitis patients an inflammatory episode was defined clinically as symptoms of either active inflammatory back pain, peripheral joint pain or enthesitis and increased fatigue. Exclusion criteria included a history of cardiovascular disease, hepatic disease, renal impairment (Creatinine >125 mmol/l), diabetes mellitus as well as pregnancy and concomitant anti-platelet use. A total of 22 consecutive patients were recruited. Patients underwent clinical review in the outpatient department and were treated according to local protocol. Anti-inflammatory medications varied from patient to patient and included the following: etanercept, methotrexate, adalimumab, tocilizumab, rituximab, tofacitinib, hydroxychloroquine, salazopyrin, sulfasalazine, abatacept, prednisolone, etoricoxib, ibuprofen, diclofenac, paracetamol, codeine. One patient received aspirin 75mg throughout the study period.

### Clinical data

Clinical data collected included age, gender, diagnosis, tender joint count and swollen joint count. Laboratory data measured included erythrocyte sedimentation rate and c-reactive protein. Patient reported outcomes were also measured to include patient reported outcome of pain, patient reported outcome of disease activity, patient reported outcome of quality of life and health assessment questionnaire.

### Blood samples

Blood samples were taken at the initial consultation at which point patients had active disease. After disease improvement, a second blood sample for platelet analysis was taken. Phlebotomy for the second sample occurred over a time period of 2–10 months post initial sampling. Timing was based on the clinical progression of the patient. Blood sample collection occurred in the morning time and at the same time as standard of care bloods. A blood volume of 2mls was collected in a standard citrate tube and was the second sample taken in the order of draw. A 21g needle was used for phlebotomy and blood was drawn gently and inverted slowly in the tube after collection to prevent damage to cells. Samples were transported to the laboratory at the Royal College of Surgeons in Ireland within 2 hours for processing. Blood samples were discarded after analysis had been performed and results obtained.

A unique previously developed assay was used to rapidly and reliably measure the interaction of platelets with surface immobilised VWF under arterial shear. In arterial circulation platelets tether to VWF via Glycoprotein Ib (GPIb) receptor. As this receptor binds to VWF, the shear forces of the flowing blood stretch it. This stimulates the platelets to initiate a complex signalling pathway and in turn leads to the activation of the Glycoprotein IIbIIIa (GPIIb/IIIa) receptor. Cross-linking of platelets occurs, leading to thrombosis. By measuring platelet behaviour from the initial capture via GPIb to the final common pathway of GPIIb/IIIa activation and stable platelet adhesion, changes in platelet function are reflected by the differences in the motion parameters measured.

### Preparation of parallel-plate flow chambers

Blood was perfused through custom-made parallel-plate flow chambers coated with VWF under arterial shear (1,500 s−1) initially described by Kent et al. [9]. Preassembled, single-use microfluidic chambers were custom-designed to consist of a 25x55mm polymethyl-methacrylate top plate (Ensinger Plastics, Mid Glamorgan, UK) fitted with inbuilt 1/16-inch polypropylene inlet and outlet connectors, a polyester gasket defining the flow path and coated on both sides with acrylic adhesive (AdhesivesResearch, Limerick, Ireland), and a microscope coverslip (24x50mm; VWR, Darmstadt, Germany). The double-sided adhesive gasket was used to seal the assembled chamber and provide a uniform flow path of 50μm x 2mm x 35mm. The channel of the parallel-plate flow chamber was coated with an aqueous solution of 100μg/mL VWF. The channel was washed three times with PBS, blocked with 1% (w/v) bovine serum albumin for 1 hour at room temperature, and washed three times with PBS prior to blood perfusion.

### Image analysis

**Fig 1:**
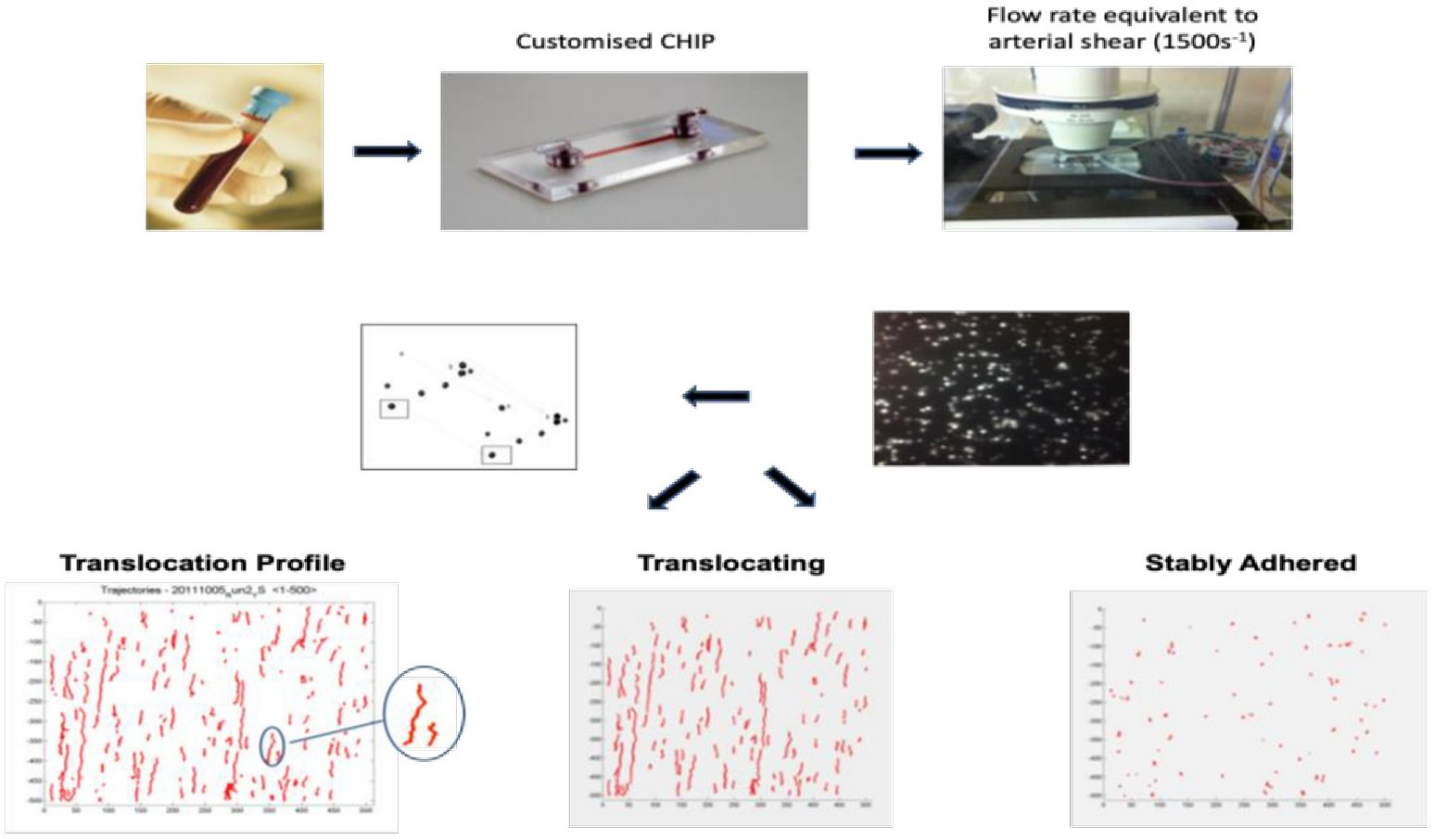
Schematic representation of dynamic platelet function assay.

Multiparameter measurements of platelet interactions with the immobilised VWF surface were recorded by digital-image microscopy and analysed using custom-designed platelet-tracking software as described by Cowman et al. [6] (see Fig 1). The MATLAB Image Processing Toolkit (version 7.12.0 R2001a) detects the position of each platelet in an x and y plane against a continuously changing background. Platelet tracks are constructed as each individual platelet’s movement is tracked from one frame to the next. Each track is further developed by assigning the original platelet to a platelet position in the next frame in order to form a weighted distance matrix. A weighted distance matrix is generated between the platelet track’s current position and other neighbouring platelets on the frame. It gives preference to platelet movement in the direction of flow over cross-stream or up-stream movement. The result is a list of platelet tracks corresponding to the associated positions over time for each platelet in an image sequence. The assay measured:

1. Total number of platelets interacting with VWF, (platelet tracks)
2. Total number of platelets translocating across VWF (GPIb dependent), (nTrans)
3. The median speed and average distance for platelets that roll, (median speed and median translocation distance)
4. The stable adhesion of individual platelets (GPIIb/IIIa dependent), (nStatic)
5. The percent of the viewing window covered by platelets, as measured at frame 500, (surface coverage)
6. The rate at which platelets first adhere to the surface, (adhesion rate)

Thus, the assay measured the physiological behaviour of platelets from the initial phase of platelet adhesion to activation and finally aggregation.

Processing of the runs started automatically and was performed without human intervention. This process took approximately five minutes which is a significant development from previous manual, error prone workflow. Data was stored on a secure web-based portal which allowed ease of access to DPFA results from multiple sites. This makes the system suitable for near patient care.

### Statistical analysis

Platelet parameters at baseline and following clinical improvement were compared using Wilcoxon signed rank test and paired student t-test. Changes in platelet function were correlated to inflammatory disease markers by Pearson Correlation.

## Results

18 of 22 patients completed the study. Four patients did not complete the study either due to laboratory or sample errors e.g. insufficient sample or loss to follow up. Of the 18, 8 had a confirmed diagnosis of seropositive rheumatoid arthritis, 1 had seronegative rheumatoid arthritis, 6 had psoriatic arthritis and the remaining 3 presented with ankylosing spondylitis. The gender distribution was 10 female patients and 8 male patients. The average age of patients at the date of first testing was 52 ±13 years, age range 30–74 years Table 1.

**Table 1:** Patient demographics.

Patient Demographics	All	Male	Female
Number	18	8	10
**Diagnosis**			
Rheumatoid Arthritis	9	2	7
Ankylosing Spondylitis	3	2	1
Psoriatic Arthritis	6	4	2
Age (in years)	52 ±13	49 ±10	54±15

Consistent with a response to treatment an improvement was seen across multiple clinical parameters from visit 1 to visit 2. Notably Tender Joint Count (Wilcoxon signed rank test) improved between visit 1 and visit 2 (5 (3.5–7) vs 1 (0.5–1), p = 0.0032) and DAS 28 CRP also improved, (paired t-test) visit 1 vs visit 2 (4.45±1.23 vs 2.65±1.22, (-1.813±1.206), p<0.0001). TJC and DAS 28 CRP were measured in patients with rheumatoid arthritis and psoriatic arthritis alone. An improvement was also seen in patient reported outcome (PRO) scores for pain, disease activity and quality of life. PRO pain score reduced from a median of 78 to 34.5 while PRO disease activity reduced from a median of 70 to 32.5 Table 2.

**Table 2:** Clinical parameters.

Clinical Parameters:	Visit 1		Visit 2	
	Median	IQR	Median	IQR
RF	80	58		
Anti-CCP	219	256		
Tender Joint Count	5	3.5	1	0.5
Swollen Joint Count	4	1.5	1	1
ESR	23	28	12	21
CRP	6	18.5	4	7
DAS 28 ESR	.02	2.05	2.36	1.75
DAS 28 CRP	4.12	1.96	2.28	1.19
PRO Pain	78	30	34.5	41.75
PRO Disease activity	70	41	32.5	40
PRO Quality of Life	70	46	30	38.5
HAQ	1.19	1.28	0.75	1

**Fig 2:**
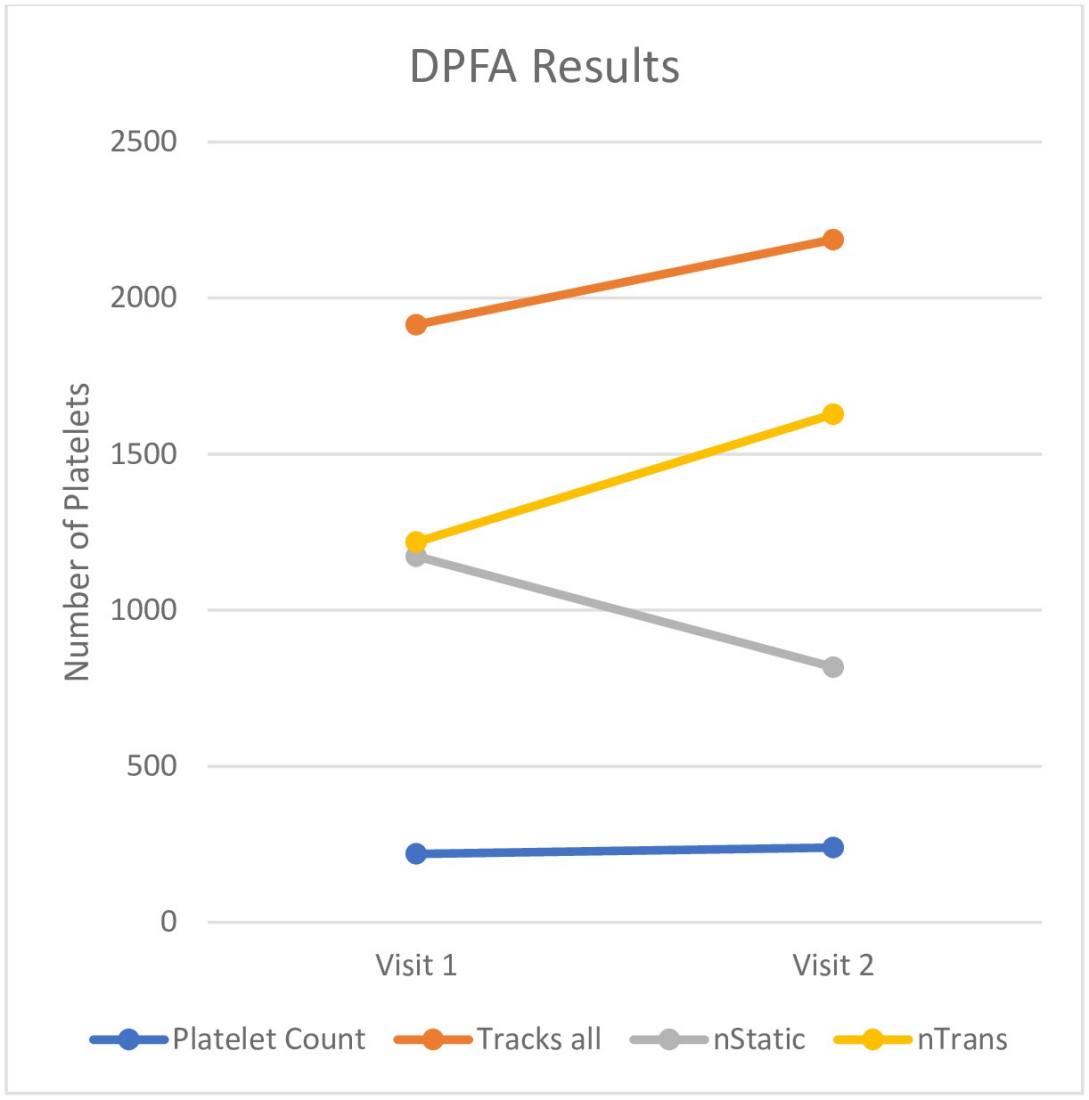
DPFA results.

Changes were also observed in dynamic platelet function Table 3. From visit 1 to visit 2 a reduction was seen in platelet adhesion with corresponding increase in platelet motion. A reduction in platelet adhesion was reflected by a decrease in FrStat, the fraction of all platelets that were stably adhered for the duration of the recording. A mean difference of -0.2152 ±0.2653 (p = 0.0031) was seen. The corresponding increase in platelet motion was represented by an increase in mean translocation distance seen at visit 2, (from 5.07 to 7.13 μm), mean difference 2.062 ±2.736 μm (p = 0.0053). The average number of platelet tracks did not differ before and after treatment and was 1915.31 and 2188.05 respectively, with a two-tailed p value of 0.24 (see Fig 2). This is representative of GPIb and GPIIb/IIIa bond formation with the VWF surface and while this did not change, the number of stably adherent bonds or the platelet adhesion rate was significantly reduced post adjustments in treatment. This is a measure of thrombus formation potential.

**Table 3:** Dynamic platelet function assay results.

Dynamic Platelet Function	Visit 1		Visit 2		p-Value
	Mean	Std Dev	Mean	Std Dev	
Platelet Count	219.17	±78.59	239.47	±87.29	
Haematocrit	34.67	±3.59	38.73	±4.71	0.0070
Tracks all	1915.31	±571.79	2188.06	±804.64	ns
nStatic	1173.40	±353.74	818.20	±379.18	0.0065
nTrans	1217.97	±382.76	1628.33	±753.64	ns
Fraction Stably Adherent	0.63	±0.13	0.41	±0.21	0.0031
Mean Translocation Distance (μm)	5.07	±1.65	7.13	±2.31	0.0053
Median Translocation Velocity (μm/s)	4.09	±2.77	4.77	±1.76	ns
%Surface Coverage	1.55	±0.41	2.15	±1.22	ns
Surface Covered End	1.66	±0.40	2.27	±1.15	ns
Stat Adhesion(s^-1^)	1.91	±0.78	1.71	±1.02	ns

Tender joint count (TJC) correlated with platelet adhesion. This was seen when comparing mean TJC with the percentage change in platelet static adhesion from visit 1 to visit 2 (Pearson r = 0.566, p = 0.028). CRP reduction correlated with velocity of platelet movement (Pearson r = 0.563, p≤0.01) indicating that changes in clinical markers of inflammation were linked to changes in platelet function.

Subgroup analysis was performed on patients prescribed non-steroidal anti-inflammatory drugs (NSAIDs). 39 per cent of patients (n = 7) were prescribed NSAIDs at the time of the first study visit. This included one patient who remained on Aspirin throughout the study period. Patients were prescribed these medications if clinically indicated. When comparing NSAID and non NSAID subgroups there was no statistically significant difference in the degree of change from baseline and following clinical improvement across the variables mean translocation distance, fraction of all platelets stably adherent (FrStat), and static adhesion of individual platelets (nStatic). From a clinical perspective the two subgroups yielded similar results. Examining the subgroup of patients prescribed NSAIDs the median Tender Joint Count improved from 5 to 1 between study visit 1 and 2. A similar reduction was seen in Swollen Joint Count (3 to 0). As in the entire study population, an improvement in median scores was seen across all PRO measurements.

## Discussion

A number of studies have highlighted the association with inflammatory arthritis and increased cardiovascular risk. According to a national longitudinal cohort study conducted in Denmark, rheumatoid arthritis is associated with the same risk of myocardial infarction as diabetes mellitus [9]. Women with rheumatoid arthritis have twice the risk of myocardial infarction compared to those without [10]. There is also an increased risk of congestive cardiac failure, asymptomatic coronary heart disease and sudden cardiac death [10]. Patients with ankylosing spondylitis have an increased risk of cardiovascular events compared to controls, with an increased prevalence of dyslipidaemia, hypertension and atherosclerotic plaques [1].

Increased platelet reactivity where platelets have an exaggerated response to a pro-inflammatory agonist is a well-known risk factor for myocardial infarction in patients with coronary artery disease. It is not clear how platelets behave in patients with inflammatory arthropathies before and after treatment. Platelet activation plays a key role in thrombosis, endothelial dysfunction and cardiovascular events. Inflammation, such as that seen with inflammatory arthropathies, causes activation of platelets via lipid mediators such as platelet activating factor (PAF), cytokines such as IFN-γ, IL-2 and chemokines such as CXCL12 and CCL22 as well as oxidative stress mechanisms that accompany inflammation and result in phospholipase A2 activation and the generation of PAF and other arachidonic acid metabolites that can also activate platelets [11]. It is proposed that by dampening this inflammatory cascade and reducing the activation of platelets, cardiovascular risk could in turn be reduced. Indeed, the QUEST-RA study showed that prolonged exposure to agents including methotrexate, sulfasalazine, glucocorticoids, leflunomide and biologics were associated with a reduction in cardiovascular morbidity following adjustment for traditional risk factors and country of origin [12]. We can extrapolate from this research that reduced platelet activity is a potential mechanism for reduced cardiovascular risk. The precise role of platelets as they behave in the arterial circulation has not been evaluated in patients with inflammatory arthropathies following treatment.

Systemic inflammation in conditions such as rheumatoid arthritis, psoriatic arthritis and ankylosing spondylitis are associated with higher cardiovascular morbidity and mortality. It is well established that inflammation activates platelets and that platelet-derived mediators in turn potentiate the inflammatory response [13]. Platelets play a crucial role in thrombosis and the pathogenesis of atherothrombosis leading to vascular complications [14]. Dynamic interaction of platelets with VWF is central in platelet adhesion. There are relatively few studies examining platelet adhesion to the vascular wall under conditions of generalised systemic inflammation secondary to autoimmune disease. Through the use of novel DPFA technology we have found that platelet adhesion was significantly reduced in patients with active inflammatory arthritis following standard procedure treatment. This supports our hypothesis that treatment of inflammatory arthritis reduces inflammation and in turn reduces platelet activation.

This study has also shown that improvements in clinical markers of inflammation are associated with statistically significant changes in platelet function. Previous studies have also alluded to this, with one study showing that after treatment of active rheumatoid arthritis and ankylosing spondylitis there was a reduction in the mean platelet volume, suggesting a change in platelet response [15]. The platelet adhesion changes we have observed support the theory that reduction in systemic inflammation reduces platelet activity. We have seen a correlation between reduced platelet activity and disease activity measured in patient reported outcomes.

Despite the well-known risk of cardiovascular death in patients with inflammatory arthritis there are no recognised guidelines on the use of antiplatelet therapy [16]. With regards to cardiovascular risk management in patients with rheumatoid arthritis, the general recommendation is to treat patients according to the national guidelines [17]. The American Heart Association guidelines recommend aspirin for primary prevention of coronary artery disease in patients with a ten-year risk of coronary heart disease of ≥10 percent [18]. The use of aspirin in addition to other NSAIDs or steroids which this patient cohort may be receiving intermittently or in the long term is an area which would benefit from further research.

This study was limited by small sample size taken from a heterogenous case-mix of inflammatory disease from a single centre. Subgroup analysis based on diagnosis was not meaningful due to the small sample size. Validated disease activity scores such as PASDAS and DAPSA, which play an important role in clinical research were not routinely collected as part of this study. Patients were recruited from routine clinical practice where such disease activity scores are not routinely measured at our centre. Further studies should be undertaken in more homogeneous disease populations using validated disease activity measures specific for each disease. Despite this critique we were able to demonstrate significant correlation between platelet reactivity and other measures of disease activity.

Sample processing was restricted by the need to transport samples off site for analysis and to process them within four hours of phlebotomy. On site dynamic platelet flow measurement would facilitate processing and would allow for increased patient recruitment. Further analysis assessing for changes in platelet function based on anti-inflammatory medication use would also be of interest.

Improvements in clinical markers of inflammation are associated with statistically significant changes in platelet function. Platelet hyper-reactivity is a potentially modifiable risk factor for cardiovascular disease in inflammatory arthritis. This novel technology allows us to develop an understanding of thrombotic pathways. Through this research we have studied platelet function in inflammatory arthritis. Larger and more wide scale studies would allow for more in-depth characterisation of platelet function. This technology could be applied widely to understand thrombotic pathways in many conditions such as COVID-19, vasculitis such as Takayasu arteritis and giant cell arteritis as well as myocardial infarction.

## Supporting information

S1 Data (XLSX)
